# On-Pump Beating/Non-Beating CABG in Stable Angina Have Similar Outcomes

**DOI:** 10.21470/1678-9741-2017-0161

**Published:** 2018

**Authors:** Victor Dayan, Juan Jose Paganini, Alvaro Marichal, Daniel Brusich

**Affiliations:** 1 Centro Cardiovascular Universitario, Hospital de Clinicas, Universidad de la Republica Oriental del Uruguay. Montevideo, Uruguay.; 2 Instituto Nacional de Cirugía Cardíaca (INCC), Montevideo, Uruguay.

**Keywords:** Coronary Artery Bypass, Cardiopulmonary Bypass, Angina, Stable

## Abstract

**Objective:**

On pump beating/non-beating coronary artery bypass grafts (CABG) has been compared in patients with unstable angina and/or severe left ventricular dysfunction. There is scarce evidence regarding the beneficial use of on-pump beating CABG in patients with stable angina and normal left ventricular function. Our aim was to study the postoperative results using both techniques in this group of patients.

**Methods:**

One thousand one hundred and forty-five patients with stable angina underwent on-pump isolated CABG in Uruguay from 2011 to 2015. Patients were grouped into beating/non-beating CABG. Operative mortality and long-term survival were evaluated as primary outcome. Logistic regression analysis was performed to define the predictive role of aortic cross clamp (AXC) on prolonged inotropic support, ventilator support and intraoperative glycemia.

**Results:**

Among the included patients, 988 underwent aortic cross clamp. No differences were found in operative mortality, stroke and long-term survival among both groups. Patients without AXC showed higher intraoperative values of glycemia and higher incidence of postoperative prolonged mechanical ventilator support (7.6% *vs.* 2.4%; *P*=0.001). The need for prolonged inotropic support was lower in this group of patients (27.4% *vs.* 49.5%; *P*<0.001).

**Conclusion:**

On-pump beating CABG has similar operative mortality and long-term survival compared with conventional AXC. Higher intraoperative glycemia and higher incidence for prolonged mechanical ventilator is associated with on-pump beating CABG. On the contrary, higher incidence for prolonged inotropic support is associated with AXC. Taking these factors into consideration, both techniques are safe and allow the surgeon to choose the most comfortable option.

**Table:** 

Abbreviations, acronyms & symbols	
AMI	= Acute myocardial infarction
AXC	= Aortic cross clamp
CABG	= Coronary artery bypass grafting
CK-MB	= Creatine phosphokinase MB isoenzyme
CPB	= Cardiopulmonary bypass
EuroSCORE	= European system for cardiac operative risk evaluation
ITA	= Internal thoracic artery
LAD	= Left anterior descending artery
PVD	= Peripheral vascular disease

## INTRODUCTION

The most common and widely used method for coronary artery bypass grafting (CABG) involves the use of cardiopulmonary bypass (CPB) with aortic cross clamp (AXC) and cardioplegic arrest. This technique allows the surgeon a still and bloodless surgical field in which construction of distal coronary anastomosis is easy and safer. Nonetheless, the use of CPB carries several disadvantages such as systemic inflammatory reaction and risk for cerebral emboli. Therefore, several groups support the use of CABG without CPB alleging better short and long-term outcomes compared with patients with CPB. Data from the most recent randomized control study has shown that although both techniques show differences in short-term outcomes (more bleeding, renal failure and respiratory complications in patients with CPB; decreased risk for early revascularization in CPB group) no differences were found in long-term outcomes between both techniques^[1,2]^.

In spite using CPB, some groups have advocated not using AXC. Some of the arguments for not using AXC are: non-uniform distribution of cardioplegia and therefore myocardial damage^[3]^; higher risk for cardiac failure specially in unstable high-risk patients^[4,5]^ and in patients with left ventricular dysfunction^[6]^. Nonetheless, the only randomized control study in this subject found higher risk of new irreversible myocardial injury in patients who underwent CABG without AXC^[7]^.

Although much has been written comparing both techniques (CPB with and without AXC), most of these studies refer to a high-risk cohort involving either unstable angina or left ventricular failure.

The objective of this study was to evaluate immediate postoperative results and long-term benefit on survival of patients with stable angina who underwent on-pump beating CABG compared with arrested heart.

## METHODS

Institutional review board from the Instituto Nacional de Cirugía Cardíaca (Montevideo, Uruguay) approved the study. Patient data was provided by the Fondo Nacional de Recursos (Resources National Fund). This Fund is the governmental entity in charge of financing all cardiac surgery procedures in Uruguay (private and public). As such, this agency has the responsibility of registering and following all procedures performed in our country. Between January 2011 and December 2015, 1145 (988 with AXC) patients underwent on-pump isolated CABG surgery for chronic ischemic heart disease. During this time-frame, the National registry included data from 18 active surgeons. Informed consent for surgical procedure was obtained in every case.

### Conventional CABG

CPB was established with aortic cannulation and bicaval venous drainage. Heparin was administered at a dose of 300 IU/kg to achieve a target activated clotting time greater than 450 s. Systemic temperature was kept between 32º and 34ºC. The aorta was cross-clamped, and myocardial protection was achieved with intermittent antegrade and retrograde crystalloid cardioplegia (Buckberg or Custodiol solution). The distal anastomoses were constructed with running sutures of 7-0 polypropylene, and the proximal anastomoses were connected to the ascending aorta with 6-0 polypropylene sutures using partial aortic cross-clamp.

After the patient was weaned from CPB and decannulated, the heparin was reversed with protamine infusion (1/1.5 rate).

### On-pump Beating CABG

The CPB circuit was the same as that for conventional CABG. The operation was continued with the assisted beating heart. The temperature of patients was kept approximately 36ºC without cooling (normothermic). The distal anastomoses were constructed before the proximal anastomoses in most cases. The left anterior descending artery (LAD) was revascularized first with the internal thoracic artery (ITA). Regional myocardial immobilization and positioning was achieved with a suction stabilizer (Octopus and Starfish, Medtronic). During anastomoses, target vessel homeostasis was obtained with temporary occlusion of the proximal coronary artery or intracoronary shunt, and/or a humidified carbon dioxide blower was used for better visualization. Distal anastomoses were made with running sutures of 7-0 polypropylene. The proximal anastomoses were created with 6-0 polypropylene sutures under a partial occlusion clamp in all cases. After weaning from CPB and decannulation, the heparin was reversed with protamine infusion (1/1.5 rate relative to heparin).

### Definitions

Operative mortality was defined as death during 30 days after surgery or during initial admission^[8]^. Prolonged inotropic support and prolonged mechanical ventilation was defined as requirement of either for more than 12h^[9]^. Perioperative acute myocardial infarction (AMI) was defined as the appearance of new Q-waves or a marked loss of R-wave forces and peak creatine phosphokinase MB isoenzyme (CK-MB) fractions greater than five times basal values^[10]^.

Categorical variables were expressed as absolute values (%), comparison between them were performed using Chi square or Fisher exact test. Continuous variables are expressed as mean±SD and comparisons were performed using Student T test.

Baseline [age, gender, diabetes, hypertension, peripheral vascular disease, smoker, creatininemia, previous CABG, previous angioplasty, chronic obstructive pulmonary disease, renal failure, number of diseased coronary vessels, ejection fraction, recent AMI, European system for cardiac operative risk evaluation (EuroSCORE)] variables along with beating/non-beating heart were evaluated by univariate analysis for each of the dependent variables studied (intraoperative glycemia, prolonged inotropic support and prolonged ventilatory support). Those which resulted significant with a *P*<0.1 were entered in the multivariate regression. Survival was evaluated with Kaplan-Meier and log-rank test used to compare survival between the groups. Logistic and linear multivariate regression analysis were performed using the enter method. Variables with a *P*<0.1 in the univariate analysis were entered into the model. Cox regression was used to evaluate predictors for long-term survival.

## RESULTS

Basal demographic characteristics are shown in Table 1. Variables were similar among patients with and without AXC except for higher incidence of peripheral vascular disease (PVD) (15.3% *vs.* 6.7%) and previous stroke (4.5% *vs.* 1.8%) in patients without AXC. Almost all patients had three vessel disease and more than 30% of patients in each group had left main disease.

**Table 1:** Demographic characteristics of the population (n=1145).

	AXC (988)	No AXC (157)	*P*
Age (SD)	65.1 (9.1)	63.9 (8.3)	0.162
Female (%)	274 (27.7)	42 (26.8)	0.798
HTN (%)	807 (81.7)	138 (87.9)	0.057
Smoker (%)	347 (35.1)	61 (38.9)	0.364
Diabetes NIR (%)	295 (29.9)	53 (33.8)	0.324
Diabetes IR (%)	45 (4.6)	8 (5.1)	0.764
Creatinine (mg/dl SD)	1.04 (0.51)	1.10 (1.03)	0.414
Previous CABG (%)	5 (0.5)	1 (0.6)	0.833
Previous PCI (%)	146 (14.8)	18 (11.5)	0.271
COPD (%)	86 (8.7)	10 (6.4)	0.327
Renal failure (%)	26 (2.6)	2 (1.3)	0.306
LVEF (SD)	55.5 (10.3)	54.4 (11.01)	0.244
LMCA (%)	345 (34.9)	50 (31.8)	0.452
Vessel disease			0.270
1	21 (2.2)	4 (2.6)	
2	160 (16.4)	16 (10.4)	
3	791 (81.3)	134 (87.0)	
Recent AMI (%)	55 (5.6)	6 (3.8)	0.366
PVD (%)	66 (6.7)	24 (15.3)	<0.001
Previous Stroke (%)	18 (1.8)	7 (4.5)	0.036*
EuroSCORE (SD)	2.98 (3.84)	2.57 (2.25)	0.200

* *P*<0.05.

AMI=acute myocardial infarction; AXC = aortic cross clamp; CABG=coronary artery bypass grafts; COPD=chronic obstructive pulmonary disease; HTN=hypertension; IR=insulin requirement; LMCA=left main coronary artery; LVEF=left ventricular ejection fraction; NIR=non-insulin requirement; PCI=percutaneous coronary intervention; PVD=peripheral vascular disease

Intra and postoperative outcomes are shown in Table 2. CPB time was slightly longer in patients with AXC (93.4±26.7 min *vs.* 81.6±22.2; *P*<0.001). Patients without AXC showed higher intraoperative values of glycemia (1.99±0.69 *vs*. 1.77±0.51; *P*<0.001) and higher incidence of postoperative prolonged ventilatory support (7.6% *vs.* 2.4%; *P*=0.001). Nonetheless, the need for prolonged inotropic support was lower in this group of patients (27.4% *vs.* 49.5%; *P*<0.001). No differences were found in operative mortality or stroke between both groups.

**Table 2:** Intra-and postoperative outcomes.

	AXC (988)	No AXC (157)	*P*
Number of bypass	3.1 (0.8)	2.9 (0.8)	0.140
LIMA (%)	954 (96.6)	147 (93.6)	0.076
BIMA (%)	122 (12.3)	17 (10.8)	0.588
CBP (min)	93.4 (26.7)	81.6 (22.2)	<0.001*
XC (min)	54.1 (17.1)		
Highest IO glycemia (mg/dl)	1.77 (0.51)	1.99 (0.69)	<0.001*
Prolonged inotropic support (%)	489 (49.5)	43 (27.4)	<0.001
Stroke (%)	11 (1.1)	2 (1.3)	0.832
Neurological dysfunction (%)	15 (1.5)	4 (2.5)	0.348
Prolonged ventilator support (%)	24 (2.4)	12 (7.6)	0.001*
ICU stay (days)	3.3 (3.9)	3.1 (2.9)	0.471
Perioperative AMI (%)	17 (1.7)	0 (0)	0.098
Hemodialysis (%)	9 (0.9)	2 (1.3)	0.665
Operative mortality (%)	37 (3.7)	4 (2.5)	0.453

AMI=acute myocardial infarction; AXC=aortic cross clamp; BIMA=bilateral internal mammary artery; CPB=cardiopulmonary bypass; IO=intra-operative; ICU=intensive care unit; LIMA=left internal mammary artery; XC=cross clamp

**Fig. 1:**
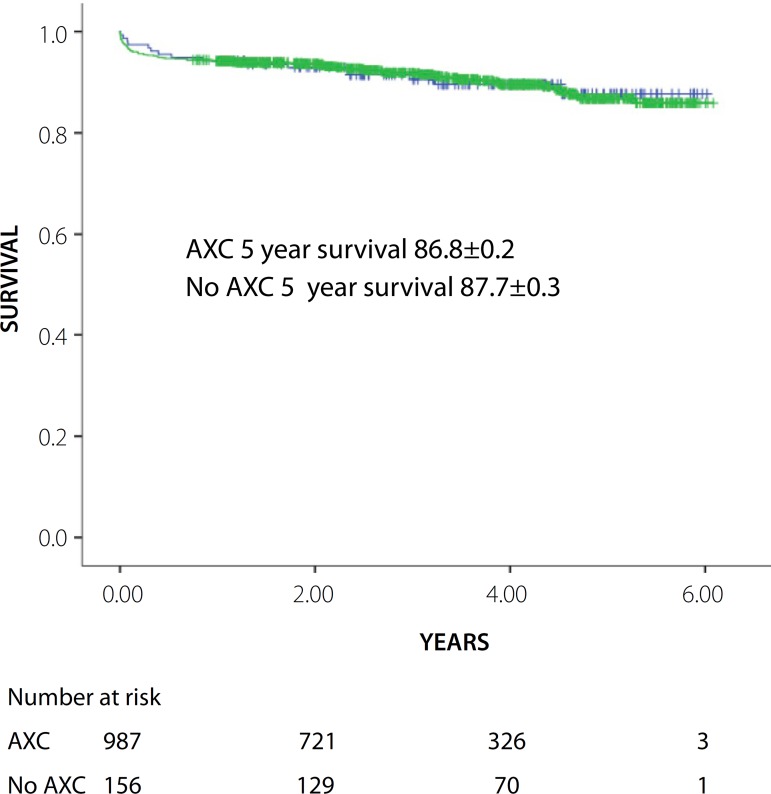
Overall survival for patients with stable angina who underwent isolated on-pump CABG. Green line - Aortic cross clamp. Blue line - beating heart. AXC=Aortic cross clamp

Long-term survival for AXC and non AXC was similar (5-year survival 86.8±0.2 *vs.* 87.7±0.3%; *P*=0.340) (Figure 1).

In order to adjust for baseline differences between groups, logistic regression analysis was performed to define the predictive role of AXC on prolonged inotropic support, ventilator support, intraoperative glycemia, operative mortality and survival.

Independent predictors for intraoperative glycemia were: diabetes (non-insulin requirement and insulin requirement), CPB time, and AXC (protective) (Table 3). Along with CPB time and EuroSCORE, AXC was found to be an independent predictor for prolonged postoperative inotropic support (Table 4). Regarding predictors for prolonged ventilatory support: AXC was protective and higher EuroSCORE was associated with increased risk (Table 5). Operative mortality and survival were not influenced by AXC.

**Table 3:** Predictors for intraoperative glycemia.

	B coefficient (95%CI)	*P*
NIR Diabetes	0.23 (0.16;0.30)	<0.001
IR Diabetes	0.35 (0.22;0.48)	<0.001
CPB time	0.003 (0.002;0.004)	<0.001
AXC	-0.24 (-0.32;-0.16)	<0.001

AXC=aortic cross clamp; CPB=cardiopulmonary bypass; IR=insulin requirement; NIR=non-insulin requirement

**Table 4:** Independent predictors for prolonged inotropic support.

	HR (95%CI)	*P*
AXC	2.19 (1.47;3.25)	<0.001
CBP time	1.009 (1.003;1.014)	0.004
EuroSCORE	1.07 (1.01;1.13)	0.020

AXC=aortic cross clamp; CBP=cardiopulmonary bypass

**Table 5:** Independent predictors for prolonged ventilation support.

	HR (95%CI)	*P*
EuroSCORE	1.08(1.01;1.16)	0.029
AXC	0.23 (0.10;0.50)	<0.001

AXC=aortic cross clamp

## DISCUSSION

Almost all data in the literature comparing on-pump beating and non-beating CABG is focused on a high-risk population either due to the presence of unstable angina or left ventricular dysfunction. There is very limited data regarding what to do in patients with stable angina. Our results show that although some differences were noted between both techniques in the immediate postoperative period, operative mortality and long-term survival is similar.

Various studies have shown that off-pump CABG is a good alternative technique for high risk patients with comparable results to on-pump CABG^[1,2,11,12]^. Some argue that off-pump CABG is a highly demanding surgical technique leading more frequently to incomplete revascularization resulting in higher incidence of short term revascularization procedures^[1,12]^. Nonetheless, the presence of diffuse atherosclerotic disease of the ascending aorta imply high risk for stroke and embolic complications with the use of AXC rendering off pump an attractive alternative.

There is evidence that with conventional CABG the arrested heart may not be as well protected from ischemia as the beating-heart technique^[13]^. This major drawback of AXC CPB surgery is most important in unstable patients and patients with left ventricular dysfunction. Several retrospective studies have shown the beneficial use of on-pump beating heart CABG in these patients^[4-6]^. In patients with end stage coronary artery disease, it has been proposed that myocardial protection with AXC is detrimental and leads to hemodynamic failure in these patients^[14]^. Furthermore, in patients with severe left ventricular dysfunction, myocardial protection with AXC has been shown to be associated with worse short-term outcomes^[5]^.

However, the only randomized control study performed in this group of patients has shown quite the opposite^[7]^. Patients with severe left ventricular dysfunction randomized to on-pump beating CABG showed higher incidence of new irreversible myocardial and on six months follow-up, only patients with AXC demonstrated an improvement in ventricular geometry. The most likely mechanism proposed was inadequate coronary perfusion to distal myocardial territories in patients with severe proximal coronary disease^[7]^.

In patients with stable angina and normal ejection fraction, our results show that both techniques are comparable regarding operative mortality and long-term survival. We have shown that patients with on-pump beating CABG have higher intraoperative values of glycemia. It is well-known the association between acute coronary syndromes and hyperglycemia^[15]^. Higher glycemia in this group of patients could therefore be explained by the ischemic burden associated with on-pump beating CABG. AXC was associated with prolonged inotropic support. Concomitantly, patients who underwent AXC had longer CPB time. Although the predictive role of AXC for prolonged inotropic support derives from multivariate logistic regression, it is difficult to separate the influence of longer CPB times exerted by this group of patients. Similar to our findings, other authors have also shown CPB to be an important predictor for prolonged inotropic support^[16]^. Although AXC showed to be a predictor for prolonged inotropic support, it has an opposite effect on prolonged mechanical ventilation. This could be explained by the increased ischemic burden exerted by on-pump beating CABG which therefore derive in diastolic dysfunction and pulmonary edema. Previous data has shown similar ultrastructural abnormalities in patients who underwent beating and arrested heart procedures^[17]^. Nonetheless, even though logistic regression aims at correcting baseline differences, patients with AXC were less sicker and this could contribute to our findings. Although our findings are supported by the only randomized control trial published up to date, in order to confirm these mechanistic explanations, further research into circulating myocardial damage markers and lung function (arterial blood gases) parameters are required.

Despite these postoperative differences, on-pump beating CABG is reliable and acceptable in patients with stable coronary artery disease and normal left ventricular function.

### Limitations

The present study is a retrospective analysis of our nation registry. Therefore, it is subject to selection bias and subject to heterogeneity among health institutions. The latter refers to the fact that some institutions promote one technique over the other and therefore their results are biased. Furthermore, since our data is extracted from the National database, indications or reasons for choosing one technique over the other one is not present. Similarly, information regarding ascending aorta calcification although very valuable is not present in the National database. Unfortunately, no data regarding myocardial damage markers and arterial blood gases were registered. Therefore, solid mechanistic explanations for the differences in prolonged mechanical ventilatory and inotropic support cannot be stated. Patency of grafts could not be evaluated since routine angiography is not performed after CABG.

## CONCLUSION

On-pump beating CABG has similar operative mortality and long-term survival compared with conventional AXC. Higher intraoperative glycemia and higher incidence for prolonged mechanical ventilation is associated with on-pump beating CABG. On the contrary, higher incidence for prolonged inotropic support is associated with AXC. Taking these factors into consideration, both techniques are safe and allow the surgeon to choose for the most comfortable option.

**Table:** 

Authors' roles & responsibilities	
VD	Substantial contributions to the conception or design of the work; or the acquisition, analysis, or interpretation of data for the work; drafting the work or revising it critically for important intellectual content; agreement to be accountable for all aspects of the work in ensuring that questions related to the accuracy or integrity of any part of the work are appropriately investigated and resolved; final approval of the version to be published
JJP	Agreement to be accountable for all aspects of the work in ensuring that questions related to the accuracy or integrity of any part of the work are appropriately investigated and resolved; final approval of the version to be published
AM	Agreement to be accountable for all aspects of the work in ensuring that questions related to the accuracy or integrity of any part of the work are appropriately investigated and resolved; final approval of the version to be published
DB	Agreement to be accountable for all aspects of the work in ensuring that questions related to the accuracy or integrity of any part of the work are appropriately investigated and resolved; final approval of the version to be published
